# Effect of preoperative injection of carbon nanoparticle suspension on the outcomes of selected patients with mid-low rectal cancer

**DOI:** 10.1186/s40880-016-0097-z

**Published:** 2016-04-04

**Authors:** Xing-Mao Zhang, Jian-Wei Liang, Zheng Wang, Jian-tao Kou, Zhi-Xiang Zhou

**Affiliations:** Department of Hepatobiliary Surgery, Beijing Chaoyang Hospital, Capital Medical University, Beijing, 100021 P.R. China; Department of Gastrointestinal Surgery, Cancer Hospital, Chinese Academy of Medical Sciences & Peking Union Medical College, 17 Panjiayuan Nanli Chaoyang District, Beijing, 100021 P.R. China

**Keywords:** Carbon nanoparticle suspension, Rectal cancer

## Abstract

**Background:**

Carbon nanoparticles show significant lymphatic tropism and can be used to identify lymph nodes surrounding mid-low rectal tumors. In this study, we analyzed the effect of trans anal injection of a carbon nanoparticle suspension on the outcomes of patients with mid-low rectal cancer who underwent laparoscopic resection.

**Methods:**

We collected the data of 87 patients with mid-low rectal cancer who underwent laparoscopic resection between November 2014 and March 2015 at Cancer Hospital, Chinese Academy of Medical Sciences & Peking Union Medical College. For 35 patients in the experimental group, the carbon nanoparticle suspension was injected transanally into the submucosa of the rectum around the tumor 30 min before the operation; 52 patients in the control group underwent the operation directly without the injection of carbon nanoparticle suspension. We then compared the operation outcomes between the two groups.

**Results:**

In the experimental group, the rate of incomplete mesorectal excision was lower than that in the control group, but no significant difference was found (2.9% vs. 7.7%, *P* = 0.342). The distance between the tumor and the circumferential resection margin was 5.8 ± 1.4 mm in the experimental group and 4.8 ± 1.1 mm in the control group (*P* = 0.001). The mean number of lymph nodes removed was 28.2 ± 9.4 in the experimental group and 22.7 ± 7.3 in the control group (*P* = 0.003); the mean number of lymph nodes smaller than 5 mm in diameter was 10.1 ± 7.5 and 4.5 ± 3.7, respectively (*P* < 0.001). Three patients in the experimental group received lateral lymph node resection. Among the three patients, we retrieved three nodes (one stained node) from the first patient, three nodes (two stained nodes) from the second patient, and two nodes (no stained nodes) from the third patient.

**Conclusions:**

Injecting a carbon nanoparticle suspension improved the outcomes of patients who underwent laparoscopic resection for mid-low rectal cancer; it also improved the accuracy of pathologic staging. Moreover, for selected patients, this technique narrowed the scope of lateral lymph node dissection.

## Background

Colorectal cancer is one of the leading causes of death in China, and owing to people’s lifestyle changes its incidence in China is increasing [1, 2]. For most patients with resectable rectal cancers (stages I, II, and partial III), surgical operation offers the best potential for cure and long-term survival. Several factors, such as the status of the circumferential resection margin (CRM), the completeness of the mesorectum, and the number of lymph nodes retrieved, have been shown to be prognostic factors of rectal cancer [3–6], whereas the effect of lateral lymph node dissection on prognosis has not been determined [7–10]. Since 1988, Yoshida et al. [11] have reported that carbon nanoparticles, which are useful, non-toxic, lymphatic tracers, have high lymphatic tropism and can reside for long periods of time in the lymph nodes. Submucosal injection of a carbon nanoparticle suspension around the tumor may be associated with operation outcomes and rectal cancer prognosis [12]. Between November 2014 and March 2015, for selected patients with mid-low rectal cancer, we performed submucous injections of carbon nanoparticle suspensions immediately prior to laparoscopic resection. We then evaluated the effect of the injection on the operation outcomes and on postoperative pathologic analyses.

## Methods

### Patients

Between November 2014 and March 2015, 87 mid-low rectal cancer patients underwent laparoscopic resection at Cancer Hospital, Chinese Academy of Medical Sciences & Peking Union Medical College. For all patients, definite diagnosis was made by colonoscopy with biopsy before the operation. Routine preoperative evaluation included physical examination, abdominal computed tomography scan, abdominal ultrasound, and barium enema. Patients who had received neoadjuvant therapy were excluded from this study.

Of the 87 patients, 35 received transanal injection of carbon nanoparticle suspensions before the operation and were assigned to the experimental group; the remaining 52 patients who did not receive transanal injection of carbon nanoparticle suspensions were assigned to the control group. Whether a patient received transanal injection of a carbon nanoparticle suspension prior to the operation was based exclusively on the patient’s individual decision after providing informed consent detailing the method and risks. Thirty-two patients in the experimental group and all 52 patients in the control group underwent traditional laparoscopic-assisted resection. For three patients in the experimental group, swollen lymph nodes close to the internal iliac vessels were detected by abdominal computed tomography scan. These three patients with clinical pathologic stage cT3N + M0 disease refused neoadjuvant therapy and underwent laparoscopic rectal cancer resection with lateral lymph node dissection.

For patients in both groups, we recorded the completeness of the mesorectum excision, the rate of positive CRM, the distance between the tumor and the CRM, the number of lymph nodes removed, and the number of lymph nodes smaller than 5 mm in diameter and compared the results.

### Surgical procedure

All patients underwent routine bowel preparation prior to surgery. After general anesthesia was administered, all patients in the experimental group then underwent disinfection of the rectal lumen. Next, 5 mL of carbon nanoparticle suspension were injected transanally to the submucosa of the rectum around the tumor 30 min before the operation. All operations were performed by the same experienced surgeons using the laparoscopy-assisted technique, following the radical resection principle. Ligation of the inferior mesenteric artery pedicle, dissociation of the intestinal canal, separation of the sacrum anterior, and dissection of the lymph nodes were completed laparoscopically. Transection of the rectum was performed through a small abdominal incision. The tissues were then removed, and the bowel was prepared for anastomosis.

Patients who underwent abdominoperineal resection did not receive an abdominal incision. All procedures, including closure of the pelvic peritoneum, were completed laparoscopically, and tissues were removed via the perineal incision.

For patients in the experimental group, the periphery of staining tissues within the tumor was the demarcation line when separating the sacrum anterior, and the stained region was protected from damage. The lymph nodes were identified by three experienced pathologists.

### Statistical analysis

Statistical analyses were performed using the SPSS software, version 16.0 (IBM, Chicago, IL, USA). *P* values less than 0.05 were considered statistically significant. Categorical variables were analyzed by the Chi-square test; continuous variables were analyzed by the Student’s *t* test.

## Results

### General parameters of patients

Patients in the two groups were well matched in terms of demographic data, including age, sex, body mass index, American Society of Anesthesiologists physical status score, concomitant diseases, tumor size, distance of tumor from the anal verge, and operation type (Table 1).

**Table 1:** Comparison of clinicopathologic characteristics in two groups of patients with mid-low rectal cancer

Parameter	Experimental group (*n* = 35)	Control group (*n* = 52)	*P* value
Sex			0.968
Men	16	24	
Women	19	28	
Age (years)^a^	60.0 ± 10.7	58.9 ± 9.4	0.631
BMI (kg/m^2^)^a^	23.6 ± 2.9	23.2 ± 2.8	0.505
ASA score			0.448
I	4	3	
II	28	41	
III	3	8	
Concomitant diseases			0.948
Yes	11	16	
No	24	36	
Tumor size (cm)^a^	4.9 ± 2.0	4.6 ± 2.3	0.619
Distance from anal verge (cm)^a^	5.6 ± 1.9	5.3 ± 1.7	0.549
Operation type (cases)			0.459
Anterior resection	28	38	
Abdominoperineal resection	7	14	
TNM stage			0.896
I	3	4	
II	6	11	
III	26	37	
Tumor differentiation			0.602
Well	2	1	
Moderate	29	46	
Poor	4	5	
Pathologic type			0.721
Adenocarcinoma	33	48	
Mucinous adenocarcinoma	2	4	

Tumor size is the largest diameter under macroscopic examination

*BMI* body mass index, *ASA* American society of anesthesiologists

^a^ These values are presented as mean ± standard deviation; other values are presented as the number of patients

### Pathologic results

The rate of incomplete mesorectal excision in the experimental group was lower than that in the control group (2.9% vs. 7.7%), although no significant difference was found (*P* = 0.342). There were no cases with positive CRM in the two groups. The distance between the tumor and the CRM was 5.8 ± 1.4 mm in the experimental group (median, 6.5 mm) and 4.8 ± 1.1 mm in the control group (median, 4.0 mm); the difference between the two groups was significant (*P* = 0.001).

**Fig. 1:**
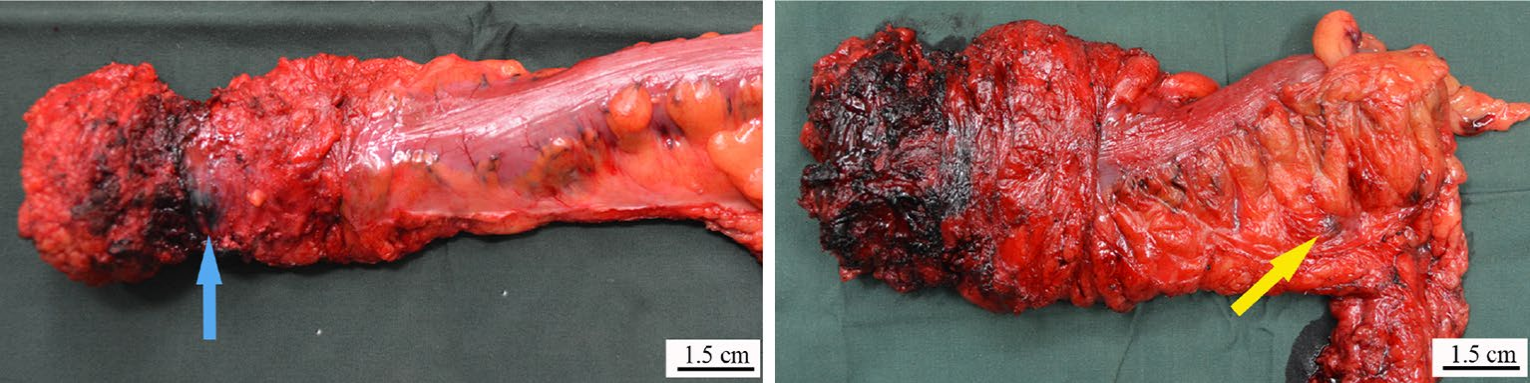
Gross specimen of rectal cancer removed under laparoscopy after injection of carbon nanoparticle suspension. The *left* shows the stained periphery which should be protected from damage during the operative procedures; the *right* shows the stained lymph node. The *blue arrow* indicates the interface of the resection; the *yellow arrow* indicates the dyed lymph node

**Fig. 2:**
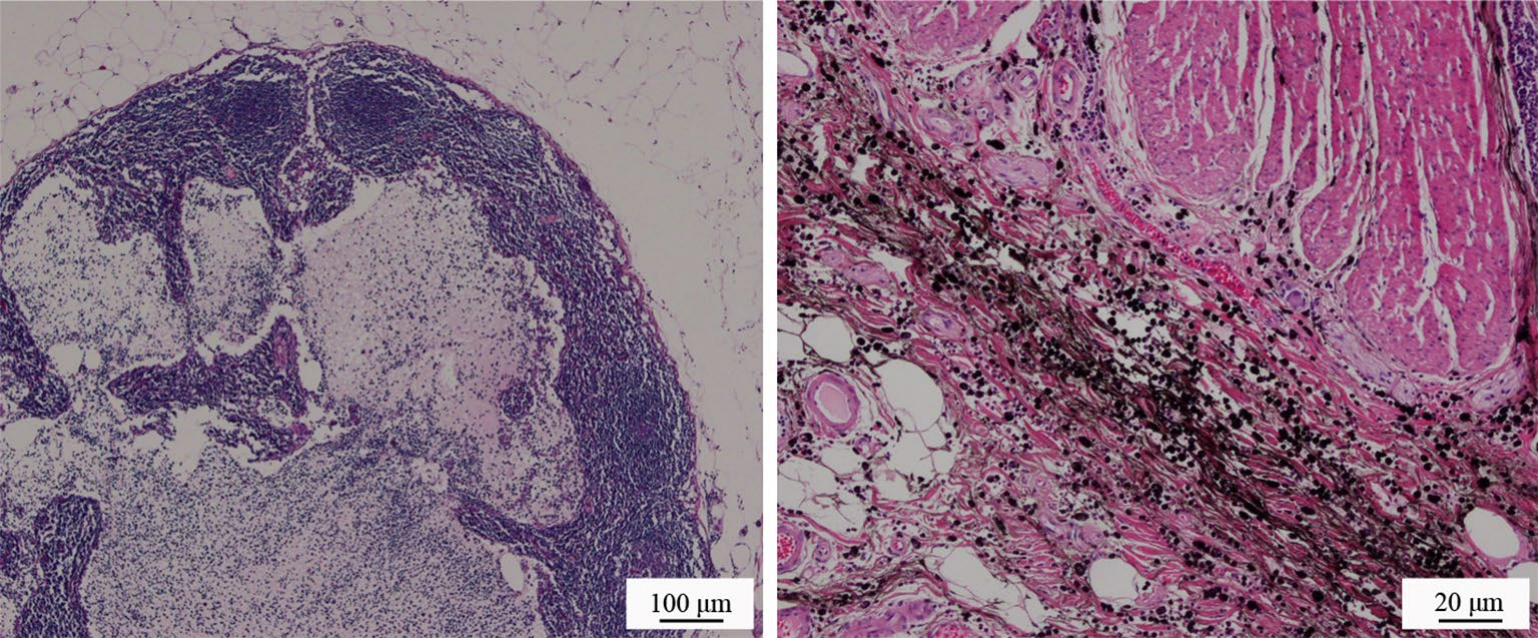
Histochemical appearance of the tumor tissues from a rectal cancer patient injected with carbon nanoparticle suspension. Tumor cells could be examined clearly under microscopy after being stained by hematoxylin and eosin, indicating that the carbon nanoparticles had no effect on the histochemical observation and diagnosis

The mean number of lymph nodes removed from patients in the experimental group was 28.2 ± 9.4 (median, 27), which was more than that from patients in the control group (22.7 ± 7.3; median, 22) (*P* = 0.003). The mean number of lymph nodes smaller than 5 mm in diameter was 10.1 ± 7.5 (median, 10) in the experimental group and 4.5 ± 3.7 (median, 4) in the control group, with a significant difference (*P* < 0.001) (Table 2). Three patients in the experimental group underwent rectal resection with lateral lymph node dissection; a total of eight lymph nodes (3, 3, and 2, respectively, from the first, second, and third patients) close to the internal iliac vessels were removed, including three lymph nodes (1, 2, and 0, respectively) that were stained by carbon nanoparticles (Figs. 1, 2). Of the eight lymph nodes close to the internal iliac vessels, cancer cells were found in two lymph nodes (0, 1, and 1, respectively)..

**Table 2:** Comparison of oncologic outcomes in the two groups of patients with mid-low rectal cancer

Outcome	Experimental group (*n* = 35)	Control group (*n* = 52)	*P* value
Number of removed LNs^a^	27 (28.2 ± 9.4)	22 (22.7 ± 7.3)	0.003
Number of LNs with diameter <5 mm^a^	10 (10.1 ± 7.5)	5 (4.5 ± 3.7)	<0.001
Completeness of mesorectum (cases)			0.342
Yes	34	48	
No	1	4	
Distance between tumor and the CRM (mm)^a^	6.5 (5.8 ± 1.4)	4.0 (4.8 ± 1.1)	0.001

*LN* lymph node, *CRM* circumferential resection margin

^a^ Values are presented as median (mean ± standard deviation)

### Others

No patients in the experimental group experienced adverse effects from the injection of the carbon nanoparticle suspension. For patients with mid-low rectal cancer, Submucosal injection of carbon nanoparticle suspensions around the tumor prior to laparoscopic resection improved the outcomes of the operation and made the pathologic staging more accurate.

## Discussion

Total mesorectal excision (TME) is the “gold standard” of surgical treatment of rectal cancer [13]. The widespread use of TME has caused the local recurrence rate of rectal cancer to decrease considerably [14]. Completeness of the mesorectum is a necessary component of TME. We found that the technique of injecting carbon nanoparticle suspensions might help surgeons identify the anatomical spaces during laparoscopic resection procedures. The interface can be identified about 30 min after the injection of carbon nanoparticle suspensions; the stained periphery is the demarcation line. The sacrum anterior can be separated through the tissue space. We found that the rate of incomplete mesorectum was reduced due to the implementation of this technique, although the results were not significant.

Lymph node involvement is an important prognostic factor for colorectal cancer patients before distant metastasis occurs. Fewer lymph nodes removed may result in worse outcomes for patients with node-positive colorectal cancer because they are more likely to be misclassified as node-negative. Kotake et al. [5] found a positive association between the number of removed lymph nodes and the number of metastatic lymph nodes. The accuracy rate of postoperative pathologic analysis might be improved when more lymph nodes are removed. In our study, more lymph nodes were removed from patients in the experimental group than from patients in the control group, and the difference was greater for lymph nodes with a diameter less than 5 mm compared with lymph nodes with a diameter more than 5 mm. Pathologists may be able to identify more lymph nodes when the nodes are stained by carbon nanoparticle suspensions.

No consensus exists as yet on lateral lymph node dissection for rectal cancer [15]. A study by Fujita et al. [10] showed that rectal resection with lateral lymph node dissection had the disadvantages of longer operation time and greater blood loss, but its benefit to the patient’s prognosis was not improved compared with that of rectal resection alone. However, Park et al. [15] concluded that laparoscopic TME with lateral lymph node dissection was “safe and feasible.” Further study is needed to determine the safety and efficacy of lateral lymph node dissection. Based on the improvements in adjuvant therapy, neoadjuvant therapy may be the best treatment for rectal cancer with suspected metastasis to the lateral lymph nodes. Mid-low rectal cancer patients with suspected metastasis to the lateral lymph nodes who refuse neoadjuvant therapy should be offered TME with lateral lymph node dissection. In our study, cancer cells were detected in some swollen lateral lymph nodes removed during operation and some of these lateral lymph nodes were stained. Despite our study’s small sample size, performing lateral lymph node dissection on patients with mid-low rectal cancer and swollen lateral lymph nodes appeared to be efficacious. According to Fujita et al. [10], narrowing the scope of lateral lymph node dissection without neglecting the lymph nodes might be the best choice. Several lateral lymph nodes were stained after the submucous injection of carbon nanoparticle suspensions. This technique enabled surgeons to remove more lateral lymph nodes without extending the scope of lateral lymph node dissection.

We did not found detailed reports about the adverse effects of the injection of carbon nanoparticle suspensions on patients’ health. Carbon nanoparticle has not been reported to have mutagenicity or carcinogenesis as confirmed by van Tongeren et al. [16]; meanwhile, Magrez et al. [17] had confirmed that carbon nanoparticle showed no adverse influence on the central nervous system, cardiovascular system, or respiratory system.

## Conclusions

Submucosal injection of carbon nanoparticle suspensions may improve the success rate of TME and make pathologic staging more accurate. Moreover, for selected patients, it may help surgeons narrow the scope of lateral lymph node dissection. Considering the limitations of our study, a larger-scale study is needed to determine the effect of Submucosal injection of carbon nanoparticle suspensions on the operation outcomes in patients with mid-low rectal cancer.
